# New heuristics for phylogeny estimation under the balanced minimum evolution criterion

**DOI:** 10.1093/bioinformatics/btaf361

**Published:** 2025-06-19

**Authors:** Daniele Catanzaro, Henri Dehaybe, Raffaele Pesenti

**Affiliations:** Center for Operations Research and Econometrics, Université Catholique de Louvain, 1348 Louvain-la-Neuve, Belgium; Center for Operations Research and Econometrics, Université Catholique de Louvain, 1348 Louvain-la-Neuve, Belgium; Department of Management, Università Ca’ Foscari, I-30121 Venezia, Italy

## Abstract

**Summary:** Recent advances in the combinatorics of the *Balanced Minimum Evolution Problem* (BMEP) enabled the characterization of the mathematical properties that a symmetric integer matrix of order n≥3 must satisfy to encode the *Path-Length Matrix* of an *Unrooted Binary Tree*. This result, together with the identification of fundamental facet-defining inequalities for the convex hull of BMEP solutions, has led to an integer linear programming formulation that currently serves as the reference exact solution algorithm. Here, we show how to exploit these advances to improve the approximation algorithms for the problem. We first leverage the tight linear programming relaxation of this formulation to develop an enhanced Neighbor Joining–like heuristic. Next, we embed this heuristic into a *Beam Search* framework to further improve the quality of the solutions. Computational experiments show that the proposed algorithms outperform existing heuristics, making their use highly desirable in practice.

**Availability and implementation:**

Codes and data are available at https://github.com/HenriDeh/BME_BeamSearch.git and archived at https://zenodo.org/records/15631441 (DOI: 10.5281/zenodo.15631440).

## 1 Introduction

**Figure 1.**
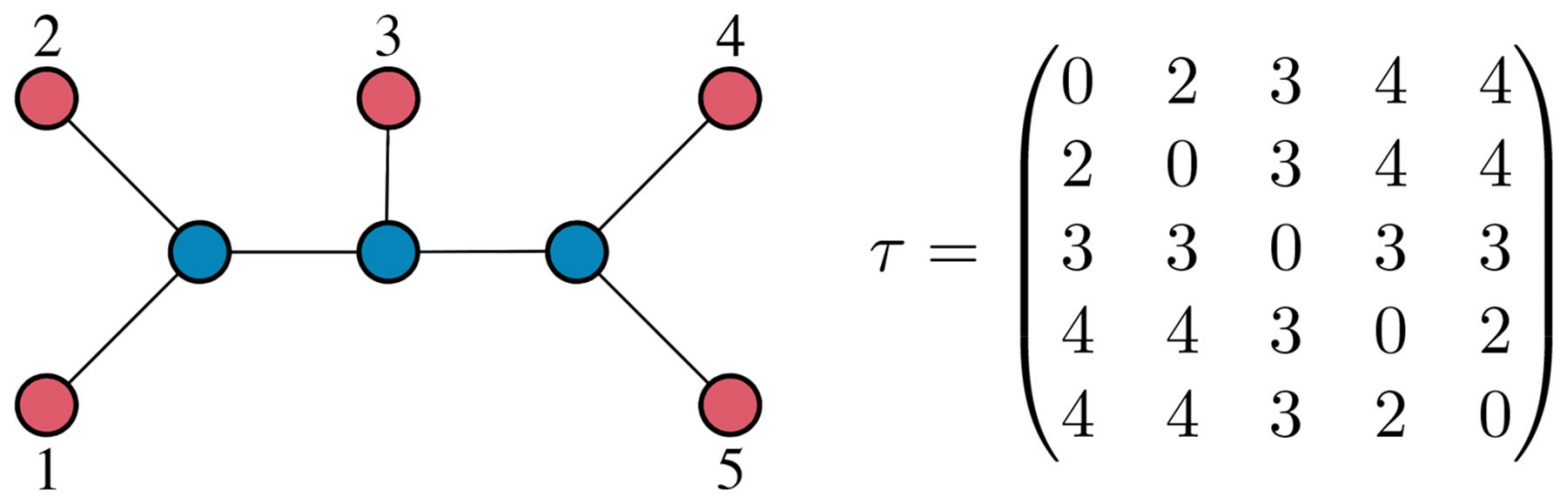
An example of a phylogeny of five taxa represented by a UBT with 5 leaves and the associated PLM τ.

Consider a set Γ={1,2,…,n} of n≥3 distinct, aligned molecular sequences, hereafter referred to as *taxa*, and a n×n symmetric matrix $D=\{d_{ij}\}$ of evolutionary distance estimates between pairs of taxa in Γ, such that $d_{ii}=0$, for all i∈Γ, and $d_{ij}>0$, for all i≠j∈Γ. Let *T* denote a *phylogeny* of Γ, i.e. an *Unrooted Binary Tree* (UBT) having Γ as set of leaves and let $U_{n}$ denote the set of the possible (2n−5)!! phylogenies of Γ (Catanzaro *et al.* 2020b). For a given phylogeny $T\in U_{n}$, let $P_{ij}^{T}$ denote the (unique) path in *T* connecting taxa i,j∈Γ and let $\tau =\{\tau_{ij}\}$ denote the *Path-Length Matrix* (PLM) associated to *T*, i.e. a n×n symmetric integer matrix, whose generic entry $\tau_{ij}\in [2,n-1]$ represents the number of edges on $P_{ij}^{T}$. For example, Fig. 1 shows a phylogeny of five taxa and the associated PLM. Finally, let $\Theta_{n}$ denote the set of PLMs associated with the phylogenies in $U_{n}$. Then, the *Balanced Minimum Evolution Problem* (BMEP) is the problem

(1) $$\underset{\tau \in \Theta_{n}}{\min}\ell (\tau )=\sum_{i\in \Gamma }\sum_{\overset{j\in \Gamma }{i\neq j}}d_{ij}2^{-\tau_{ij}}.$$

The BMEP formalizes, in terms of a nonlinear combinatorial optimization problem, the *Balanced Minimum Evolution* (BME) criterion of phylogenetic estimation, introduced in the literature on distance-based methods by Desper and Gascuel in 2004. BME originates from Rzhetsky and Nei’s *Minimum Evolution* (ME) criterion and primarily differs from ME in its use of a specific branch-length estimation model introduced by Pauplin in 2000. This model was designed to address key limitations of the classical Ordinary Least-Squares method, the most critical of which was the occurrence of negative branch lengths (see Catanzaro *et al.* 2022 for a recent historical review of BMEP). BME posits that the phylogeny of a given set of molecular sequences (taxa) is the one that minimizes the sum of their *cross-entropies* (Catanzaro *et al.* 2020a). This phylogeny is provably *statistically consistent*, i.e. it converges to the true phylogeny of taxa if the distance estimates $d_{ij}$ approximate the true evolutionary distances of taxa when considering longer and longer molecular sequences (Desper and Gascuel 2004, Gascuel 2005, Gascuel and Steel 2006). BME figures among the most accurate estimation criteria in distance-based methods (Lefort *et al.* 2015); it can be easily adapted to any kind of molecular data for which a dissimilarity measure is available (Schwartz 2019) and because it works on distance estimates between taxa rather than on their molecular sequences, it results to be computationally less demanding than alternative estimation criteria based on maximum parsimony, maximum likelihood, or Bayesian inference (Gascuel 2005). These features make BME particularly suitable for general-purpose phylogenetic analyses.

Inferring phylogenies under the BME criterion implies solving the BMEP. Unfortunately, this problem is NP-hard and inapproximable within $c^{n}$, for some constant c>1, unless P=NP (Fiorini and Joret 2012). This negative result holds even in the case where the structure of the UBT is fixed and only the assignment of the *n* taxa to the *n* leaves of the UBT needs to be identified (Frohn 2021). The BMEP can however be approximated within a factor of two if D is *metric*, i.e. if its entries also satisfy the triangle inequality (Fiorini and Joret 2012).

The pursuit of practical solution algorithms for the BMEP has driven two decades of extensive research, focusing on its combinatorial properties (Semple and Steel 2004, Pardi 2009, Pardi *et al.* 2010, Cueto and Matsen 2011, Haws *et al.* 2011), optimization aspects (Forcey *et al.* 2016, 2017, Catanzaro *et al.* 2020b), and the development of both exact solution algorithms (Pardi 2009, Catanzaro *et al.* 2022) and heuristic approaches (Gascuel 2005, Pardi 2009, Fiorini and Joret 2012, Lefort *et al.* 2015, Gasparin *et al.* 2023) to tackle its instances. Particularly relevant for this article are some recent theoretical advances—briefly summarized in the next section for the sake of completeness—that led to the characterization of the set of the PLMs of UBTs, of basic properties and facets of their convex hull, and to the development of an *Integer Linear Programming* (ILP) formulation that currently serves as the reference exact solution algorithm for the problem (see Catanzaro *et al.* 2025). Here, we show that these findings can be leveraged to design matheuristics for the BMEP, i.e. heuristic algorithms that exploit a valid mathematical programming formulation for the problem to generate high-quality approximate solutions. In particular, we show that these methods can construct phylogenies with shorter tree lengths than those produced by current state-of-the-art approaches. This article is organized as follows. In the next section, we briefly recall both some classic results on the *Tree Realization Problem* (TRP), introduced by Buneman (1974) and Waterman *et al.* (1977), and some recent results on the characterization of $\Theta_{n}$ that constitute the theoretical foundation of the ILP formulation presented in Catanzaro *et al.* (2025). In Section 3, we present the construction heuristic for the BMEP. In particular, we discuss first the core matheuristic and its rationale and then we show how it can be embedded within a Beam Search to enhance the quality of the resulting solution. In Section 4, we discuss the computational experiments obtained when running the proposed algorithms in combination with the local search method in FastME 2 (Lefort *et al.* 2015) on a number of benchmark instances of the BMEP. Finally, in Section 5, we present some open questions that may inspire future developments.

## 2 Some background on the TRP and the BMEP

Given a matrix $D=\{d_{ij}\}$ on a target set Γ of n≥3 points, the TRP asks whether there exists a *tree realization* of D, i.e. a weighted tree (T,w) having Γ as set of leaves and nonnegative branch-lengths $w=\{w_{e}\}$ satisfying the following *path-length constraint*:

(2) $$\sum_{e\in P_{ij}^{T}}w_{e}=d_{ij}\;\forall \;i<j\in \Gamma .$$

A classical result for metric distance matrices states that

Proposition 1. *(Buneman 1974)*

*There is a tree realization for a metric distance matrix* D  *on a target set* Γ  *of n points if and only if for any four distinct* i,j,p,q∈Γ  *the following four-point condition holds:*
 

(3) $$d_{ij}+d_{pq}\leq \max\left\{d_{ip}+d_{jq},d_{iq}+d_{jp}\right\}.$$

Observe that the tree that realizes a metric distance matrix D is not necessarily unrooted and binary, but can be transformed into a UBT by adding dummy nodes and edges with possibly null branch-lengths.

Determining the properties that an integer metric matrix τ must satisfy to encode the PLM of a UBT in $U_{n}$ is equivalent to determining the properties that ensure a tree realization for τ when also imposing that the weighted tree (T,w) is a UBT with branch-lengths all equal to 1. This further requirement, unfortunately, makes the four-point condition necessary to ensure a *UBT realization* but not sufficient (Catanzaro *et al.* 2025). Denoted by ${\left[\alpha ,\beta \right]}_{N}$ the interval of the integers between α and β (included), a possible characterization of a UBT realization is provided by the following result:

Proposition 2. *(* *Catanzaro et al.* 2025)

*A square matrix* τ  *of order* n≥3  *encodes the PLM of a UBT* $T\in U_{n}$  *if and only if it satisfies*
 

(4) $$\tau_{ii}=0\;\;\forall \;i\in \Gamma$$

 

(5) $$\tau_{ij}\in {\left[2,n-1\right]}_{N}\;\;\forall \;i<j\in \Gamma$$

 

(6) $$\tau_{ji}=\tau_{ij}\;\;\forall \;i<j\in \Gamma$$

 

(7) $$\tau_{1i}+\tau_{1j}\geq 2+\tau_{ij}\;\;\forall \;\text{distinct}\;i,j\in \Gamma \setminus \{1\}$$

 

(8) $$\tau_{1i}+\tau_{ij}\geq 2+\tau_{1j}\;\;\forall \;\text{distinct}\;i,j\in \Gamma \setminus \{1\}$$

 

(9) $$\sum_{\overset{j\in \Gamma }{j\neq i}}2^{-\tau_{ij}}=\frac{1}{2}\;\;\forall \;i\in \Gamma$$

*and, for all distinct* j,p,q∈Γ∖{1}*, exactly one of the following* strong four-point conditions:

(10a) $$\tau_{1j}+\tau_{pq}+2\leq \tau_{1p}+\tau_{jq}=\tau_{1q}+\tau_{jp}$$

 

(10b) $$\tau_{1p}+\tau_{jq}+2\leq \tau_{1j}+\tau_{pq}=\tau_{1q}+\tau_{jp}$$

 

(10c) $$\tau_{1q}+\tau_{jp}+2\leq \tau_{1j}+\tau_{pq}=\tau_{1p}+\tau_{jq}.$$

Conditions (4)–(6) impose that τ must be a symmetric matrix with null diagonal and integer nondiagonal entries in [2,n−1]. Conditions (7) and (8) impose τ to be metric. Conditions (9) impose *Kraft’s equalities* which ensure the degree constraint on the internal nodes of the weighted tree (Catanzaro *et al.* 2025). Finally, Conditions (10) impose Buneman’s four-points conditions, that ensure the connectivity and acyclicity of the weighted tree. Proposition 2 allows to reformulate the BMEP in terms of an ILP problem. Specifically, let $L={\left[2,n-1\right]}_{N}$ denote the set of values that can be taken by a generic path-length $\tau_{ij}$. In addition, let $x_{ij}^{\ell }$ denote a decision variable equal to 1 if and only if $\tau_{ij}=\ell \in L$, and let $y_{pq}^{j}$ denote a decision variable equal to 1 if and only if the subtree containing 1 and *j* is disjoint from that containing *p* and *q*. Then, the following formulation is valid for the BMEP (Catanzaro *et al.* 2025):

(11a) $$\begin{array}{cc} \min & \sum_{i\in \Gamma }\sum_{j\in \Gamma \setminus \{i\}}d_{ij}\left(\sum_{\ell \in L}2^{-\ell }x_{ij}^{\ell }\right) \end{array}$$

 

(11b) $$\begin{array}{cc} s.t. & \sum_{\ell \in L}x_{ij}^{\ell }=1\;\forall \;i\neq j\in \Gamma \end{array}$$

 

(11c) $$\begin{array}{c} x_{ij}^{\ell }=x_{ji}^{\ell }\;\forall \;i\neq j\in \Gamma ,\;\ell \in L \end{array}$$

 

(11d) $$\begin{array}{c} \tau_{ij}=\sum_{\ell \in L}\ell x_{ij}^{\ell }\;\forall \;i\neq j\in \Gamma \end{array}$$

 

(11e) $$\begin{array}{c} \sum_{j\in \Gamma \setminus \{i\}}\sum_{\ell \in L}2^{-\ell }x_{ij}^{\ell }=\frac{1}{2}\;\forall \;i\in \Gamma \end{array}$$

 

(11f) $$\begin{array}{c} \tau_{1i}+\tau_{1j}-\tau_{ij}\geq 2\;\forall \;i\neq j\in \Gamma \setminus \{1\} \end{array}$$

 

(11g) $$\begin{array}{c} \tau_{1i}+\tau_{ij}-\tau_{1j}\geq 2\;\forall \;i\neq j\in \Gamma \setminus \{1\} \end{array}$$

 

(11h) $$\begin{array}{c} y_{pq}^{j}+y_{jq}^{p}+y_{jp}^{q}=1\;\forall \;\text{distinct}\;j,p,q\in \Gamma \setminus \{1\} \end{array}$$

 

(11i) $$\begin{array}{c} \tau_{1j}+\tau_{pq}\geq 2(1-y_{jp}^{q})+\tau_{1p}+\tau_{jq}-(2n-2)y_{pq}^{j}\\ \;\forall \;\text{distinct}\;j,p,q\in \Gamma \setminus \{1\} \end{array}$$

 

(11j) $$\begin{array}{c} \sum_{i\in \Gamma }\sum_{j\in \Gamma \setminus \{i\}}\sum_{\ell \in L}\ell 2^{-\ell }x_{ij}^{\ell }=2n-3 \end{array}$$

 

(11k) $$\begin{array}{c} x_{ij}^{\ell }\in \{0,1\}\;\forall \;i\neq j\in \Gamma ,\;\ell \in L \end{array}$$

 

(11l) $$\begin{array}{c} y_{pq}^{j}\in \{0,1\}\;\forall \;\text{distinct}\;j,p,q\in \Gamma \setminus \{1\} \end{array}$$

 

(11m) $$\begin{array}{c} \tau_{ij}\in L\;\forall \;i\neq j\in \Gamma . \end{array}$$

The objective function (11a) of the ILP formulation for the BMEP linearizes (1). Constraints (11 b) impose that for each distinct pair of taxa i,j∈Γ, the path-length $\tau_{ij}$ takes precisely one value in *L*. Constraints (11c) impose the symmetry condition on path-lengths $\tau_{ij}$. Constraints (11d) tie $\tau_{ij}$ variables to $x_{ij}^{\ell }$ variables. Constraints (11e) impose (9). Constraints (11f) and (11 g) impose (7) and (8). Constraints (11 h) and (11i) impose Buneman’s four-points conditions (10). Constraint (11j) impose that path-lengths lie on a particular manifold known as *UBT manifold* (Catanzaro *et al.* 2020b, 2025). Finally, constraints (11k)–(11m) impose the integrality of all variables. A more compact ILP formulation can be obtained by declaring $x_{ij}^{\ell }$ variables only for all i,j∈Γ such that i<j and substituting $\tau_{ij}$ variables with the right-hand side of (11d). Here, we prefer to keep them for the sake of clarity. Formulation (11) is characterized by $O(n^{3})$ variables and constraints. A Branch-&-Cut algorithm based on it exactly solves BMEP instances containing up to 32 taxa within the reference time of 1 hour and in the computational environment described in Catanzaro *et al.* (2025). For larger instances, however, this algorithm may prove too slow for practical use. Hence, in the next sections we leverage the very tight lower bounds provided by the *linear programming* relaxation of Formulation (11) to approximate the optimal solution $\tau^{*}$ to BMEP instances.

## 3 Materials and methods: A matheuristic for the BMEP

### 3.1 Agglomeration methods

**Figure 2.**
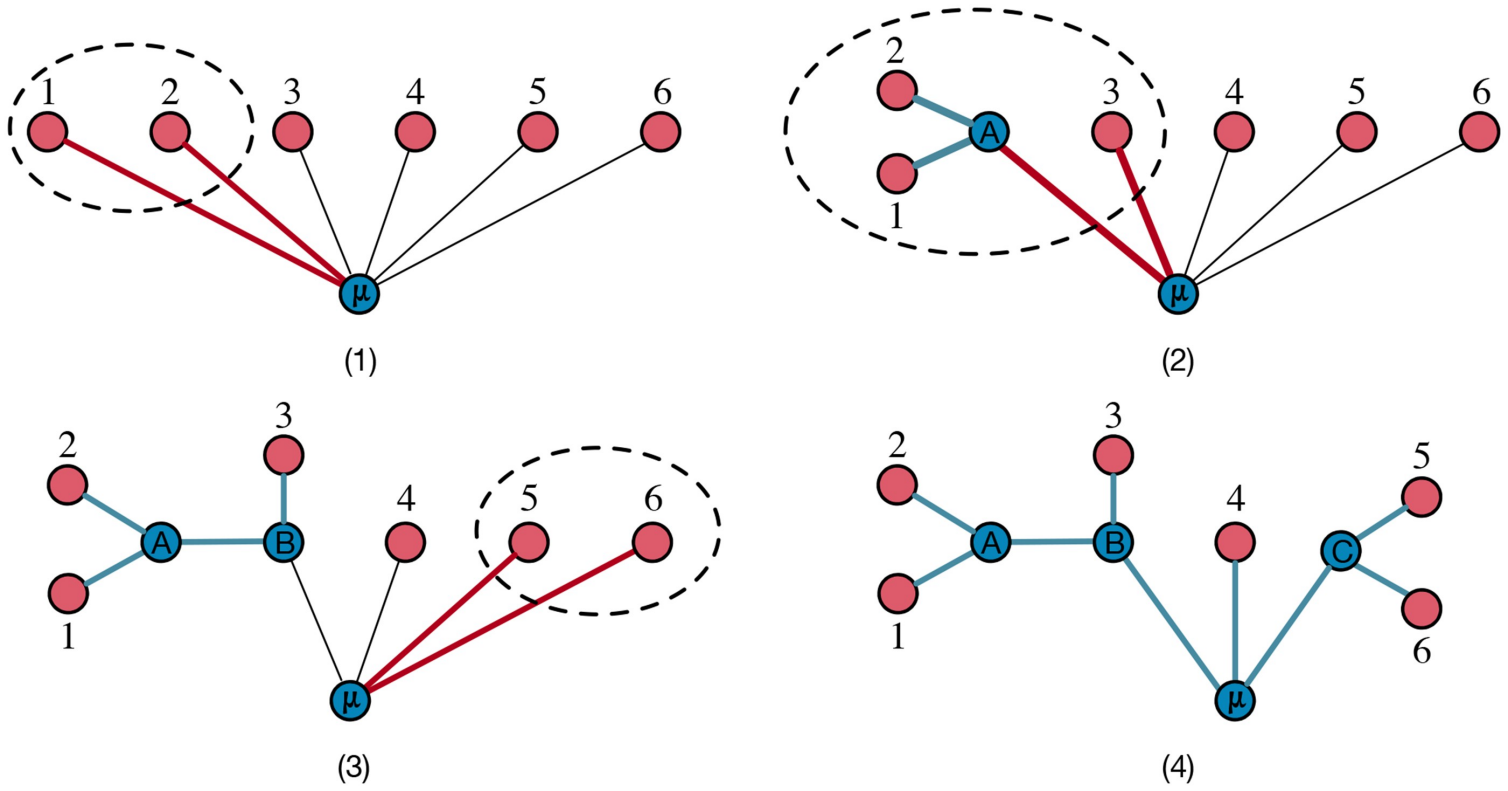
The three iterations of an agglomeration method required to generate a phylogeny of six taxa: iteration (1) merges sigletons {1} and {2} and introduces internal node *A*; iteration (2) merges cluster {1,2} with the singleton {3} and introduces node *B*; iteration (3) merges singletons {5} and {6} and introduces node *C*; and (4) resulting in the final 6-taxon phylogeny.

The matheuristic described in this section belongs to the family of *agglomeration methods* (Saitou and Nei 1987, Felsenstein 2004, Gascuel 2005, Gascuel and Steel 2006). The core idea of these methods consists of constructing first an initial star-tree constituted of a central node μ and *n* distinct clusters of taxa, each containing initially a single taxon in Γ. Then, at each step, two clusters of taxa are iteratively merged, with each merge introducing a new internal node that serves as the *common ancestor* of the merged clusters. This new internal node is linked to the central node μ, while the edges connecting μ to the common ancestors of merged taxa are removed. The process is repeated for n−3 steps, resulting in a UBT that represents the phylogeny of Γ upon termination. Figure 2 shows an example of this agglomeration method.

Agglomeration methods differ in how they select clusters to merge at each step *r*, based on the current set $N^{r}$ of common ancestor nodes and the matrix $D^{r}$ of the distances $d_{pq}^{r}$ between each pair of elements $i,j\in N^{r}$. Initially, $N^{1}=\Gamma$ and $D^{1}$ is the taxa (evolutionary) distance matrix D. At the end of step *r*, $N^{r+1}$ is set equal to $N^{r}\cup \{v\}\setminus \{p,q\}$, where *p* and *q* are the common ancestors of the two merged clusters, and *v* is the common ancestor of the cluster created by the merge; $D^{r+1}$ is derived from $D^{r}$ by a *reduction operation*, i.e. by deleting the respective *p*-th and *q*-th rows and columns and by adding a new row and column whose entries encode the distances between *v* and the remaining clusters. For example, referring again to Fig. 2, $D^{2}$ is obtained from $D^{1}$ by removing the rows and columns corresponding to taxa 1 and 2 and inserting a new row and column representing the distances from node *A* to nodes in $N^{2}=N^{1}\cup \{A\}\setminus \{1,2\}=\{A,3,4,5,6\}$. In the remainder of the article, we denote by $T^{r}$ the tree generated by the agglomeration method at step *r* and by $s_{ij}^{r}$ the *topological distance* between the leaves *i* and *j* on $T^{r}$, i.e. the number of edges on the path $P_{ij}^{r}$. Note, in particular, that when r=1, $T^{1}$ is the initial star-tree, hence $s_{ij}^{1}=2$, for all i,j∈Γ. At the end of step r=n−3, $T^{n-2}$ is a UBT in $U_{n}$, hence $s_{ij}^{r}=\tau_{ij}$. We also define the *generalized tree length formula* $\ell (T^{r})$ as (Semple and Steel 2004):

(12) $$\ell (T^{r})=\sum_{i\in \Gamma }\sum_{\overset{j\in \Gamma }{i\neq j}}\prod_{k\in V_{P_{ij}^{r}}}{\left(\delta (k)-1\right)}^{-1}d_{ij}$$

where $V_{P_{ij}^{r}}$ is the set of internal nodes in the path $P_{ij}^{r}$ and δ(k) the degree of node *k*. Note that when r=n−2, (12) is precisely the objective function of the BMEP.

The *Neighbor-Joining* (NJ) algorithm (Saitou and Nei 1987, Gascuel 2005) is an example of a possible agglomeration method which is particularly interesting in the context of the BMEP because it greedily minimizes (12) (Gascuel and Steel 2006). We recall that if *p* and *q* are the common ancestors of the two clusters that are merged at the *r*-th step of the NJ algorithm, then the values of the distances of the new node *v* from the common ancestors of the other clusters are defined by means of the following reduction:

(13) $$d_{vk}^{r+1}=d_{kv}^{r+1}=\frac{d_{pk}^{r}+d_{qk}^{r}}{2}\;\forall k\in N^{r}\setminus \{p,q\}.$$

The common ancestors *p* and *q* of the two clusters to merge at the *r*-th step are instead identified by solving the following problem:

(14) $$(p,q)=\underset{\overset{i,j\in N^{r}}{i\neq j}}{\text{argmin}}\left(|N^{r}|-2\right)d_{ij}^{r}-\sum_{k\in N^{r}}\left(d_{ik}^{r}+d_{jk}^{r}\right).$$

### 3.2 The LPNJ algorithm

Building on the notation and definitions introduced in the previous sections, we now present a novel NJ-like agglomeration method, hereafter referred to as the *Linear Programming Neighbor-Joining* (LPNJ) algorithm, that leverages the *Linear Programming* (LP) relaxation of Formulation (11) to guide the cluster selection step. We will first introduce an agglomeration method that, given a PLM, constructs the associated phylogeny. We then describe the LPNJ algorithm. Finally, we prove the equivalence between the UBT reconstruction method and a version of LPNJ that uses the optimal integer solution of Formulation (11) in place of the linear programming relaxation. This result serves as a theoretical justification for the proposed LPNJ algorithm.

#### 3.2.1 The UBT reconstruction algorithm

Consider the following agglomeration method, hereafter referred to as the *UBT Reconstruction Algorithm*, that can be used to reconstruct the UBT *T* associated to a PLM.

This algorithm operates with a matrix of topological distances τ, for example identified by the optimal integer solution of Formulation (11), instead of evolutionary distances, or, equivalently, assumes that evolutionary distances coincide with topological distances, i.e. D=τ. At each iteration, it merges two clusters whose distance $\tau_{ij}^{r}$ between the common ancestors *i* and *j* is equal to 2, and updates the distance matrix according to the following reduction operation rule

(15) $$\tau_{vk}^{r+1}=\tau_{kv}^{r+1}=\tau_{ik}^{r}-1=\tau_{jk}^{r}-1\;\forall k\in N^{r}\setminus \{p,q\}$$

where *v* is the new common ancestor of the cluster created by the merge. At the end of its execution, the UBT reconstruction algorithm generates a UBT because if $\tau^{r}$ is the PLM of a UBT, then there are at least two entries in $\tau^{r}$ such that $\tau_{ij}^{r}=2$ for $i,j\in N^{r}$. Furthermore, by the triangular inequalities (11f), if $\tau_{ij}^{r}=2$, then $\tau_{ik}^{r}=\tau_{jk}^{r}$ for all $k\in N^{r}$. Now, observe that, for all *r*, $\tau^{r}$ is the PLM of a UBT. First, $\tau^{1}=\tau$ is the PLM of a UBT by assumption. Furthermore, if $\tau^{r}$ is the PLM of a UBT *T*, then rule (15) makes $\tau^{r+1}$ be the PLM of the UBT obtained by pruning the leaves *i* and *j* and their adjacent edges from *T*. Finally, the UBT generated by the UBT reconstruction algorithm is the phylogeny associated with $\tau^{1}$, since rule (15) makes the distance between each pair *p*, *q* of leaves be equal to $\tau_{pq}^{1}$.

#### 3.2.2 The LPNJ algorithm

The UBT reconstruction algorithm described in the previous section is an agglomeration method that uses topological information (i.e. the topological distances between the nodes associated with the common ancestors of the clusters of taxa) to select the clusters to merge, as opposed to standard agglomeration methods, such as the NJ algorithm, which rely just on the evolutionary distances. The UBT reconstruction algorithm would return the optimal UBT $T^{*}$ if the optimal path length matrix $\tau^{*}$ were given.

The LPNJ algorithm that we present here leverages the features of both aforementioned agglomeration methods. Specifically:

- Like the NJ algorithm, it initially sets $D^{1}$ equal to the taxa evolutionary distance matrix and, at each subsequent step *r*, defines the distances $d_{vk}^{r+1}$ between the common ancestor *v* of the newly generated cluster and the other common ancestors $k\in N^{r+1}\setminus \{v\}$ according to rule (13).
- Like the UBT reconstruction algorithm, at each subsequent step *r*, it selects the two clusters to merge based on an estimate of the topological distances of the optimal UBT $T^{*}$ between their common ancestors *i* and *j*. Specifically, it estimates the distances between the common ancestors in $N^{r}$ by computing the matrix ${\tilde{\tau }}^{r}$, which is the solution to the LP relaxation of Formulation (11) when applied to the evolutionary distance matrix $D^{r}$. Thus, the selection operation is given by:
  (16) $$(i,j)=\underset{\overset{p,q\in N^{r}}{p\neq q}}{\text{argmin}}{\tilde{\tau }}_{pq}^{r}$$

The rationale behind the LPNJ algorithm is based on the following theorem.

Theorem 1. *Consider the following version of the LPNJ algorithm. Let* $\tau^{*r}$  *be an optimal integer solution of Formulation (11) applied to the evolutionary distance matrix* $D^{r}={\left\{d_{ij}^{r}\right\}}_{i,j\in N^{r}}$*. Apply the cluster selection rule (16) and the reduction rule (13). Then, there exists an optimal solution* $\tau^{*r+1}$  *of (11) when applied to the evolutionary distance matrix* $D^{r+1}$  *which can be derived from* $\tau^{*r}$  *using the matrix reduction rule (15) of the UBT reconstruction algorithm on* $\tau^{*r}$.

Proof. Without loss of generality assume that the LPNJ algorithm merges the clusters whose common ancestors are associated with the first and the second rows of matrix $D^{r}$. Then, $\tau_{12}^{*r}=2$ and $\tau_{1j}^{*r}=\tau_{2j}^{*r}$ for all $j\in N^{r}$. In addition, $N^{r+1}=N^{r}\cup \{v\}\setminus \{1,2\}$, where *v* represents the common ancestor of the newly formed cluster. Finally, by definition $\tau^{*r}$ is an optimal solution to the following problem (with constraints omitted for brevity).

$$\begin{array}{c} \underset{\tau }{\min}z^{*r}=\sum_{i\in N^{r}}\sum_{j\in N^{r}}d_{ij}^{r}2^{-\tau_{ij}^{r}}\\ =2d_{12}^{r}2^{-2}+\sum_{i\in N^{r}\setminus \{1,2\}}\sum_{j\in N^{r}\setminus \{1,2\}}d_{ij}^{r}2^{-\tau_{ij}^{r}}\\ \;+2\sum_{j\in N^{r}\setminus \{1,2\}}\left(d_{1j}^{r}+d_{2j}^{r}\right)2^{-\tau_{1j}^{r}}\\ =2d_{12}^{r}2^{-2}+\sum_{i\in N^{r+1}\setminus \{v\}}\sum_{j\in N^{r+1}\setminus \{v\}}d_{ij}^{r}2^{-\tau_{ij}^{r}}\\ \;+2\sum_{j\in N^{r+1}\setminus \{v\}}\frac{d_{1j}^{r}+d_{2j}^{r}}{2}2^{-(\tau_{1j}^{r}-1)}. \end{array}$$

Now, consider the optimal solutions $\tau^{*r+1}$ of the problem

$$\underset{\tau }{\min}z^{*r+1}=\sum_{\overset{i\in N^{r+1}}{i\neq v}}\sum_{\overset{i\in N^{r+1}}{j\neq v}}d_{ij}^{r+1}2^{-\tau_{vj}^{r+1}}+2\sum_{\overset{i\in N^{r+1}}{j\neq v}}d_{vj}^{r+1}2^{-\tau_{vj}^{r+1}},$$

where by hypothesis $d_{vj}^{r+1}=(d_{1j}^{r}+d_{2j}^{r})/2$ for all $j\in N^{r}$, and observe that $z^{*r}=2d_{12}^{r}2^{-2}+z^{*r+1}$ holds. Now suppose, by contradiction, that there is no solution $\tau^{*r+1}$ such that $\tau_{vj}^{*r+1}=\tau_{1j}^{*r}-1$ for all $j\in N^{r}$ while $\tau_{ij}^{*r+1}=\tau_{ij}^{*r}$ for all $i,j\in N^{r+1}\setminus \{v\}$. This fact implies that $z^{*r}\neq 2d_{12}^{r}2^{-2}+z^{*r+1}$, which leads to a contradiction. Thus, the statement follows. ▪

An immediate consequence of the above theorem is that if we were able to estimate $\tau^{*r}$ exactly at each step *r* of the LPNJ algorithm, it would return the optimal UBT $T^{*}$. In fact, only the minimal entries of ${\tilde{\tau }}^{r}$ need to be exact for LPNJ to return $T^{*}$. Although this may be not the case in general, the solution ${\tilde{\tau }}^{r}$ to the LP relaxation of Formulation (11) considered by the LPNJ algorithm constitutes a good approximation of $\tau^{*r}$ (Catanzaro *et al.* 2025).

### 3.3 A Beam Search

To improve the quality of the solution provided by the LPNJ algorithm we introduce in this section a *Beam Search* approach (Lowerre 1976) that explores multiple alternative cluster merges at each step of an agglomeration algorithm. Beam Search balances exploration and computational efficiency by maintaining a fixed number of alternatives, denoted by the beam width parameter *B*, at each level of the search.

Let $\Xi^{r}$ be the set of alternatives considered at step *r* in a Beam Search approach applied to an agglomerative method. The algorithm proceeds as follows:

- Initialization: Set $\Xi^{1}=\{T^{1}\}$, where $T^{1}$ is the initial star-tree corresponding to the taxa set (e.g. Fig. 2(1)).
- For each step  r=1,…,n−3:
  - Compute the set $C^{r}$ of all possible trees obtained from the trees in $\Xi^{r}$ by merging two clusters of taxa.
  - Set $\Xi^{r+1}$ as the set of the *B*-best trees in $C^{r}$ according to a given *ranking criterion*.
- Return: The best tree in $\Xi^{n-2}$.

We refer to the algorithms obtained by applying Beam Search to NJ and LPNJ as *BeamNJ* and *BeamLPNJ*, respectively. We will compare the performance of these algorithms for different values of *B* in Section 4.

#### 3.3.1 BeamNJ

The natural ranking criterion for selecting trees at each step in the BeamNJ method is given by the generalized tree length formula (12).

In the following, we show an efficient approach for computing the value of a tree $T^{r+1}\in C$, obtained from a tree $T^{r}\in \Xi^{r}$ by merging two clusters whose common ancestors are nodes *p* and *q*, respectively. To this end, let $\Gamma_{v}$ denote the taxa cluster whose common ancestor is $v\in N^{r}$. By (13), we obtain:

(17) $$d_{pq}^{r}=\sum_{i\in \Gamma_{p}}\sum_{j\in \Gamma_{q}}d_{ij}2^{-s_{ij}-1}.$$

This result allows us to express the tree length as

(18) $$\ell (T^{r})=\overset{⁁}{\ell }(T^{r})+\sum_{i\in N^{r}}\sum_{j\in N^{r}}{\left(|N^{r}|-1\right)}^{-1}d_{ij}^{r}$$

where

(19) $$\overset{⁁}{\ell }(T^{r})=\sum_{v\in N^{r}}\sum_{i\in \Gamma_{v}}\sum_{j\in \Gamma_{v}}d_{ij}2^{-s_{ij}^{r-1}},$$

a result that can be also easily derived from Gascuel and Steel (2006). Now, assume that *p* and *q* are the merged common ancestors at step *r*. Then, from (17) and (18) we obtain

$$\overset{⁁}{\ell }(T^{r+1})=\overset{⁁}{\ell }(T^{r})+\frac{d_{pq}^{r}}{2}$$

which leads to

(20) $$\begin{array}{c} \ell (T^{r+1})=\overset{⁁}{\ell }(T^{r})+\frac{d_{pq}^{r}}{2}\\ +\sum_{i\in N^{r}\setminus \{p,q\}}\sum_{j\in N^{r}\setminus \{p,q\}}{\left(|N^{r}|-2\right)}^{-1}d_{ij}^{r}\\ +\sum_{v\in N^{r}\setminus \{p,q\}}{\left(|N^{r}|-2\right)}^{-1}\frac{d_{vp}^{r}+d_{vq}^{r}}{2}. \end{array}$$

This expression can be rewritten as

(21) $$\begin{array}{c} \ell (T^{r+1})=\overset{⁁}{\ell }(T^{r})+\frac{d_{pq}^{r}}{2}+\\ \frac{\sum_{i\in N^{r}}\sum_{j\in N^{r}}d_{ij}^{r}-\sum_{v\in N^{r}}d_{vp}^{r}-\sum_{v\in N^{r}}d_{vq}^{r}}{2(|N^{r}|-2))}. \end{array}$$

Observe that the three terms in the numerator, each expressed as a sum, can be precomputed for each $T^{r}\in \Xi^{r}$ to avoid redundant computation. Equation (21) is used as the ranking criterion for BeamNJ. It is worth noting here that Neighbor-Joining (NJ) is mathematically equivalent to BeamNJ when B=1.

#### 3.3.2 BeamLPNJ

The optimality criterion for selecting trees at each step in the BeamLPNJ method is based on a specific underestimate of the value $\ell (T^{n-2})$ of a UBT $T^{n-2}$ that can be derived by each tree $T^{r}\in \Xi^{r}$ considered at step *r*. Specifically, we define:

(22) $$\underline{\ell }(T^{r+1})=\overset{⁁}{\ell }(T^{r})+\frac{d_{pq}^{r}}{2}+\sum_{i\in N^{r+1}}\sum_{\overset{j\in N^{r+1}}{j\neq i}}d_{ij}^{r+1}2^{-{\tilde{\tau }}_{ij}^{r+1}}$$

where, as usual, {p,q} is the selected pair of common ancestors, and ${\tilde{\tau }}^{r+1}$ is the solution to the linear programming (LP) relaxation of Formulation (11) when applied to the evolutionary distance matrix $D^{r+1}$. Equation (22) is a lower bound on the value of $\ell (T^{n-2})$ and would be an ideal ranking criterion for BeamLPNJ. Unfortunately, it requires computing ${\tilde{\tau }}^{r+1}$ for each tree in $C^{r}$. Instead, we propose to use an underestimate of $\underline{\ell }(T^{r})$, that is based solely on the solution ${\tilde{\tau }}^{r}$. We derive it based on the following rationale. By Theorem 1, if ${\tilde{\tau }}^{r}=\tau^{*r}$, then, at step *r*, selecting any tree $T^{r+1}\in C^{r}$ such that $\underline{\ell }(T^{r+1})=\ell (T^{*})$ provides the optimal selection of common ancestors to merge. In this case, it holds that

(23) $$\underline{\ell }(T^{r+1})=\underline{\ell }(T^{r})-\sum_{i\in N^{r}}d_{ip}^{r}2^{-{\tilde{\tau }}_{ip}^{r}}-\sum_{i\in N^{r}}d_{iq}^{r}2^{-{\tilde{\tau }}_{iq}^{r}}+\sum_{i\in N^{r+1}}\frac{d_{ip}^{r}+d_{iq}^{r}}{2}2^{-{\tilde{\tau }}_{ip}^{r}-1}+\frac{d_{pq}^{r}}{2}-2d_{pq}^{r}2^{-{\tilde{\tau }}_{pq}^{r}}$$

which only requires ${\tilde{\tau }}^{r}$. By (13), (23) can be simplified to

(24) $$\begin{array}{c} \underline{\ell }(T^{r+1})=\overset{⁁}{\ell }(T^{r})+\frac{d_{pq}^{r}}{2}+\sum_{i\in N^{r}}\sum_{j\in N^{r}}d_{ij}^{r}2^{-{\tilde{\tau }}_{ij}^{r}}-d_{pq}^{r}2^{-({\tilde{\tau }}_{pq}^{r}-1)}\\ =\underline{\ell }(T^{r})+\frac{d_{pq}^{r}}{2}-d_{pq}^{r}2^{-({\tilde{\tau }}_{pq}^{r}-1)}. \end{array}$$

However, in general, $\underline{\ell }(T^{r})\leq \underline{\ell }(T^{r+1})\leq \ell (T^{n-2})$, as ${\tilde{\tau }}^{r}\neq {\tilde{\tau }}^{*r}$. This choice of ranking criterion makes BeamLPNJ with B=1 a slightly different version of LPNJ where (16) is replaced by

$$(i,j)=\underset{p,q}{\text{argmin}}d_{pq}\left(\frac{1}{2}-\frac{1}{2^{{\tilde{\tau }}_{pq}-1}}\right)$$

which, like LPNJ, equally favors pairs of taxa when ${\tilde{\tau }}_{pq}=2$ but introduces the value of $d_{pq}$ in other cases.

## 4 Results and Discussion

### 4.1 Datasets

We carried out computational experiments on four sets of evolutionary distance matrices. Set A consists of the nine matrices derived from real aligned DNA datasets proposed by Catanzaro *et al.* (2006). Set B includes the RDPII and ZILLA matrices (Stamatakis 2005, Guindon *et al.* 2010) as used by Gasparin *et al.* (2023) in their experiments. Set C is a set of symmetric Random Doubly Stochastic Matrices (RDSM) with zero diagonals generated with the Sinkhorn-Knopp algorithm (Sinkhorn and Knopp 1967). Set D consists of symmetric Random Integer Matrices (RIM) with entries in the range [1…10], again excluding the diagonal, which is set to zero. The instances for the experiments were derived from Sets A and B by selecting the relative distances of the subset consisting of the first *n* taxa of each matrix, with *n* ranging from 10 to 60 (or the largest multiple of 5 for matrices with fewer than 60 taxa), incremented by 5. Datasets C and D were directly generated to include ten matrices for each considered value of *n*.

### 4.2 Experimental setup

We compare the tree lengths produced by the proposed Beam Search algorithms with those generated by the methods implemented in the state-of-the-art tree inference software, FastME 2.0 (Lefort *et al.* 2015). FastME offers five construction algorithms: NJ, BIONJ, Unweighted NJ, TaxAdd OLS, and TaxAdd BME. The trees generated by each construction method considered in the experiments are further optimized using the Subtree Pruning and Regrafting (SPR) local search method, which is also provided by FastME. In the case of BeamNJ and BeamLPNJ, the *B* resulting phylogenies undergo the local search procedure before returning the best one. In practice, even the linear relaxation of Formulation (11) can be prohibitively time-consuming to solve for medium-sized instances. To address this, we further relax the formulation by removing the Buneman conditions in constraints (11 h)–(11i) and the associated *y* variables. Additionally, we compare two variants of BeamLPNJ, one that includes the triangular inequalities (11f) and (11 g) (BeamLPNJ+tri), and one that excludes them (BeamLPNJ-Tri). All variants of the Beam Search algorithm (BeamNJ, BeamLPNJ-Tri, and BeamLPNJ+Tri) are tested for values of *B* in {1,5,10,15}. We further accelerate the solving of the linear programs by reducing the maximum path-length from n−1 to ${\;\text{log}\;}_{2}{\left(n-1\right)}^{2}$ and by adding the maximum path-length to the exponent in the objective function in order to improve the numerical stability of the solver.

### 4.3 Computational results

Table 1 shows for each dataset which methods most consistently found the best tree across all considered methods and the average optimality gaps. The gap for a single instance is calculated as the difference between the length of the tree found by the method and a lower bound on that length, divided by the lower bound. The lower bound is determined by the value of the linear programming relaxation of Formulation (11), excluding Buneman’s constraints. The detailed result data are available at https://github.com/HenriDeh/BME_BeamSearch.git. Several key observations can be drawn from the results. In general, Beam Search algorithms with beam width B>1 show superior performance in identifying solutions that are close to optimal, consistently achieving the smallest optimality gaps on average. Among the standalone agglomeration algorithms (without SPR improvements), BeamNJ emerges as the most effective choice for biological instances (datasets A and B), showing progressively better performance as the beam width *B* increases. In contrast, for synthetic datasets, the BeamLPNJ-Tri method proves to be the most efficient. Notably, the inclusion of triangular inequalities in the formulation tends to hinder the performance of BeamLPNJ methods, both in terms of solution quality and computational speed. However, when B=1, Beam Search algorithms simplify to standard greedy algorithms: in this scenario, BeamNJ becomes equivalent to the NJ algorithm, while BeamLPNJ closely resembles LPNJ. In such cases, the advantage of employing an LP-based algorithm becomes more evident, as solving the linear relaxation of the optimization model yields a valuable metric for guiding the pairwise joining process. We hypothesize that the relatively loose lower bound provided by estimate (24) is the main factor behind the declining performance of BeamLPNJ as the beam width *B* increases, while BeamNJ benefits from the exact tree length as its ranking criterion. When the phylogenies generated by the agglomeration methods are further refined using local SPR moves, BeamLPNJ methods slightly outperform BeamNJ. BeamLPNJ-Tri then appears to be a robust choice. Table 2 reports the average run times of the methods as a function of the instance sizes. Beam Search methods are, unsurprisingly, more computationally demanding. Their run time and memory usage scale linearly in *B* and that of BeamLPNJ methods also increases cubically in *n* due to the increase in formulation size.

**Table 1.** Comparison of the tested methods for each dataset.^a^

		Agglomeration								Agglomeration + SPR							
		Set A		Set B		Set C		Set D		Set A		Set B		Set C		Set D	
Method	B	Best	Gap	Best	Gap	Best	Gap	Best	Gap	Best	Gap	Best	Gap	Best	Gap	Best	Gap
BeamLPNJ+Tri	15	34.3	5.43	35.2	4.2	33.6	39.17	27.3	66.3	55.6	5.07	**85.2**	4.0	**45.5**	38.47	40.0	64.0
BeamLPNJ-Tri	15	**37.4**	5.6	31.8	4.16	**39.1**	**39.06**	**34.5**	**66.19**	57.6	5.08	83.0	**3.99**	43.6	**38.4**	**42.7**	**63.71**
BeamNJ	15	**37.4**	**5.26**	**63.6**	**4.1**	36.4	39.77	27.3	67.92	54.5	5.07	68.2	4.0	40.0	38.99	24.5	65.38
BeamLPNJ+Tri	10	33.3	5.53	33.0	4.19	24.5	39.27	27.3	66.42	56.6	5.09	73.9	4.0	37.3	38.49	30.0	64.16
BeamLPNJ-Tri	10	32.3	5.77	35.2	4.18	30.0	39.23	24.5	66.31	**58.6**	**5.06**	81.8	4.0	41.8	38.43	39.1	63.92
BeamNJ	10	33.3	5.3	43.2	4.12	32.7	39.88	20.9	68.32	54.5	5.09	65.9	4.0	38.2	39.13	23.6	65.45
BeamLPNJ+Tri	5	21.2	5.62	28.4	4.29	22.7	39.33	20.9	66.72	54.5	5.08	67.0	4.01	29.1	38.63	24.5	64.53
BeamLPNJ-Tri	5	28.3	5.72	34.1	4.2	27.3	39.28	22.7	66.81	56.6	5.09	71.6	4.01	32.7	38.67	26.4	64.41
BeamNJ	5	28.3	5.35	34.1	4.12	26.4	40.37	15.5	69.05	54.5	5.08	59.1	4.01	31.8	39.27	20.0	65.88
BeamLPNJ+Tri	1	19.2	5.61	20.5	4.28	15.5	39.94	5.5	68.73	49.5	5.11	63.6	4.02	20.9	39.31	10.0	66.11
BeamLPNJ-Tri	1	22.2	5.63	23.9	4.23	12.7	39.88	8.2	68.84	52.5	5.11	61.4	4.01	18.2	39.16	14.5	66.33
BeamNJ	1	11.1	5.67	18.2	4.23	8.2	41.81	9.1	71.72	48.5	5.1	52.3	4.03	16.4	39.92	10.0	67.29
BIONJ	–	6.1	6.33	9.1	4.39	5.5	42.21	0.0	80.99	37.4	5.2	26.1	4.19	20.0	39.82	0.9	78.58
NJ	–	11.1	5.67	18.2	4.23	8.2	41.81	9.1	71.61	48.5	5.1	52.3	4.03	16.4	39.92	10.9	67.28
TaxAdd BME	–	2.0	6.99	9.1	4.49	0.9	50.03	0.0	84.31	44.4	5.16	77.3	4.01	15.5	40.29	8.2	68.03
TaxAdd OLS	–	1.0	8.01	9.1	4.78	0.9	51.25	0.9	85.66	43.4	5.15	55.7	4.03	12.7	40.52	10.9	67.95
Unweighted NJ	–	8.1	6.43	13.6	4.41	4.5	42.29	4.5	72.81	31.3	6.13	29.5	4.26	16.4	39.9	14.5	66.9

a The *Best* columns indicate the percentage of instances in the respective datasets where the best tree across all methods was identified by the method. The *Gap* columns report the average optimality gap (in %) over the dataset. Results are presented separately for standalone agglomeration algorithms and after applying SPR optimization. Bold values highlight the best results in each column.

**Table 2.** Average run time of each method in seconds per instance size (number of taxa).

		Instance size (*n*)										
Method	B	10	15	20	25	30	35	40	45	50	55	60
BeamLPNJ+Tri	15	0.12	0.75	2.81	7.75	18.17	37.7	74.38	139.01	251.18	391.5	636.3
BeamLPNJ-Tri	15	0.07	0.39	1.4	3.45	7.12	13.04	21.99	34.81	51.84	75.62	106.34
BeamNJ	15	<0.01	0.01	0.03	0.05	0.08	0.13	0.2	0.34	0.49	0.65	0.84
BeamLPNJ+Tri	10	0.09	0.53	1.89	5.42	12.39	27.01	51.12	92.72	170.06	265.29	423.55
BeamLPNJ-Tri	10	0.05	0.28	0.95	2.45	5.07	9.27	15.37	24.16	36.91	53.14	74.96
BeamNJ	10	<0.01	<0.01	0.02	0.03	0.05	0.08	0.12	0.22	0.31	0.42	0.55
BeamLPNJ+Tri	5	0.05	0.3	1.08	2.98	7.06	13.92	26.07	47.84	84.31	125.41	206.35
BeamLPNJ-Tri	5	0.03	0.17	0.64	1.42	3.02	5.39	9.1	14.45	21.81	30.6	43.0
BeamNJ	5	<0.01	<0.01	<0.01	0.02	0.03	0.04	0.06	0.11	0.16	0.2	0.26
BeamLPNJ+Tri	1	0.02	0.12	0.42	1.0	2.11	4.13	7.05	11.25	19.03	25.64	38.1
BeamLPNJ-Tri	1	0.02	0.08	0.25	0.65	1.27	2.33	3.78	6.09	9.05	13.62	18.79
BeamNJ	1	<0.01	<0.01	<0.01	<0.01	<0.01	<0.01	0.01	0.02	0.03	0.04	0.05
BIONJ	–	<0.01	<0.01	<0.01	<0.01	<0.01	<0.01	<0.01	<0.01	<0.01	0.01	0.01
NJ	–	<0.01	<0.01	<0.01	<0.01	<0.01	<0.01	<0.01	<0.01	<0.01	0.01	0.01
TaxAdd BME	–	<0.01	<0.01	<0.01	<0.01	<0.01	<0.01	<0.01	<0.01	<0.01	<0.01	0.01
TaxAdd OLS	–	<0.01	<0.01	<0.01	<0.01	<0.01	<0.01	<0.01	<0.01	<0.01	<0.01	0.01
Unweighted NJ	–	<0.01	<0.01	<0.01	<0.01	<0.01	<0.01	<0.01	<0.01	<0.01	0.01	0.01

## 5 Conclusion

The approximation algorithms proposed in this study—based on the recent findings presented in (Catanzaro *et al.* 2025)—successfully deliver improved near-optimal solutions for instances of the BMEP that are otherwise impractical to tackle using the current state-of-the-art exact solution algorithms. However, since these methods rely on solving a linear program, the application of LPNJ and BeamLPNJ algorithms remains constrained to small instances. Further exploration of enhanced formulations and their mathematical properties could potentially extend the applicability of these approaches to larger problem instances. Moreover, the results presented in this article raise an important question: why does the inclusion of triangular inequalities in the formulation hinder the performance of BeamLPNJ algorithms? Developing tighter lower bounds for the BeamLPNJ criterion could improve the pair selection process, potentially leading to even better performance. Addressing these challenges presents an important direction for future research.
